# NVVC/NHJ Durrer prizes 2017

**DOI:** 10.1007/s12471-018-1106-4

**Published:** 2018-03-23

**Authors:** J. J. Piek

**Affiliations:** AMC Heart Center, Academic Medical Center, Amsterdam, The Netherlands

The NVVC/NHJ publication prizes are named after one of the founding fathers of Dutch Cardiology, professor D. Durrer (1918–1984) who was the chair of the department of Cardiology at the former Wilhelmina Gasthuis in Amsterdam. He was an outstanding pioneer in the field of electrophysiology with classical works on the electric activation of the heart in the 1960s and 1970s [1]. In 1972, he also founded the Interuniversity Cardiology Institute of the Netherlands (ICIN). Moreover, the Durrer Center, founded in 2008, carries his name in his memory. The Durrer Center is a national multidisciplinary collaboration of academic research institutes in the field of cardiology, genetics and biostatistics.

The Netherlands Heart Journal, the official journal of the Netherlands Society of Cardiology (NVVC), awarded the Durrer prizes to two outstanding NHJ articles published in 2017. These articles are selected based upon their originality and scientific quality as well as the number of citations. One article is basic oriented and the other article has a clinical focus. The 2 articles are selected out of a total of 114 articles published in the NHJ 2017.

The best basic-oriented article was entitled ‘Potential new mechanism of pro-arrhythmia in arrhythmogenic cardiomyopathy: focus on calcium sensitive pathways’ [2]. It is a review of the experimental evidence of calcium sensitive pathways as a cause of pro-arrhythmia in arrhythmogenic cardiomyopathy. Arrhythmogenic cardiomyopathy or its most well-known subform arrhythmogenic right ventricular cardiomyopathy (ARVC) is a cardiac disease characterised by gradual replacement of the myocardial mass by fibrous and fatty tissues, leading to dilatation of the ventricular wall, arrhythmias and progression towards heart failure. ARVC is commonly regarded as a disease of the intercalated disc in which mutations in desmosomal proteins are an important causative factor. It has been identified that the Dutch founder mutation PLN R14Del plays a key role in ARVC patients in the Netherlands. Phospholamban (PLN protein) has an important role in the regulation of sarcoplasmic reticulum calcium load, suggesting that Ca^2+^ sensitive proteins may play a key role in the maladaptive remodelling of the macromolecular protein complex that forms the intercalated disc. The authors emphasise that we need to interpret the evidence from experimental models with caution as we do not have the appropriate models that mimic the end-stage disease in arrhythmogenic cardiomyopathy patients.

The best clinical article is entitled ‘Pathophysiology and treatment of atherosclerosis: Current view and future perspective on lipoprotein modification treatment’ [3]. It is a review of the current understanding of the pathophysiology and treatment of atherosclerotic disease including future perspective on several novel classes of drugs that target atherosclerosis. The focus is on pathophysiology and the medical interventions of low-density lipoprotein cholesterol (LDL-C), high-density lipoprotein cholesterol (HDL-C), triglycerides (TG) and lipoprotein(a) (Lp(a)). The authors concluded that lowering LDL-C with standard therapy remains the cornerstone of the medical intervention and treatment of atherosclerotic disease. In high-risk patients with statin intolerance or in high-risk patients who do not obtain the desired LDL-C level with intensive statin treatment cholesterol absorption inhibitors, especially ezetimibe, should be considered. Bile acid sequestrants, fibrates and niacin are not recommended. PCSK-9 inhibitors are important agents for the treatment of dyslipoproteinaemia, but the long-term efficacy and safety need to be proven while the costs involved are of concern. The role of HDL-C modulation for the treatment of atherosclerosis is still uncertain and the results of ongoing clinical trials are awaited. Finally, a new class of molecules targeting Lp(a) shows promising efficacy and safety in early phase trials.

The first authors of these articles received an educational grant, provided by the NVVC, at the Annual Spring Meeting of the NVVC held at the congress centre Leeuwenhorst at Noordwijkerhout, the Netherlands, on 5 and 6 April 2018. The NHJ would like to congratulate the authors with these awards and thank them for submitting their excellent work to our journal. The editorial board of the NHJ hopes that the Durrer prizes act as a stimulus for authors to send their best paper to our journal.
